# Cholecystectomy After Previous Bariatric Surgery with Special Focus on Pregnant Patients—Results from Two Large Nationwide Registries

**DOI:** 10.1007/s11695-020-04409-3

**Published:** 2020-01-24

**Authors:** Jonas Hedström, Johan Nilsson, Mikael Ekelund, Roland Andersson, Bodil Andersson

**Affiliations:** 1 Department of Clinical Sciences Lund, Surgery, Lund University and Skane University Hospital, SE-221 85 Lund, Sweden; 2 Department of Clinical Sciences Lund, Cardiothoracic Surgery, Lund University and Skane University Hospital, SE-221 85 Lund, Sweden

**Keywords:** Bariatric surgery, Cholecystectomy, Complications, Gallstone disease, Pregnancy

## Abstract

**Background:**

Biliary complications during pregnancy is an important issue. The aim of this study was to examine if there is an increased risk to perform cholecystectomy during pregnancy in patients with previous bariatric surgery in comparison to other females subjected to cholecystectomy.

**Methods:**

The Nationwide Swedish Registry for Gallstone Surgery (GallRiks) and the Scandinavian Obesity Surgery Registry (SOReg) were combined. Female patients 18–45 years old were included. The study group was patients with a history of bariatric surgery whom were pregnant at the time of cholecystectomy. This group was compared with pregnant patients without previous bariatric surgery and non-pregnant with and without previous bariatric surgery.

**Results:**

In total, 21,314 patients were included and 292 underwent surgery during pregnancy. From 1282 patients identified in both registers, 16 patients were pregnant at the time of cholecystectomy. Acute cholecystectomy was performed in 5922 (28%) non-pregnant and 199 (68%) pregnant (*p* < 0.001), including 11/16 (69%) pregnant with previous bariatric surgery. When comparing all pregnant patients, those with previous bariatric surgery had longer operative time (*p* = 0.031) and length of stay (*p* = 0.043), but no differences were seen when only comparing patients with an acute indication for surgery. There was no difference in complications comparing pregnant patients with previous bariatric surgery with non-pregnant, both with and without previous bariatric surgery.

**Conclusions:**

Cholecystectomy during pregnancy in patients with previous bariatric surgery seems to be safe. The increased risk seen in the non-pregnant group after previous bariatric surgery is not seen in pregnancy, possibly due to an optimization of the circumstances at surgery.

## Introduction

Cholecystectomy is one of the most common abdominal surgical procedures worldwide, including in Sweden, where an annual number of about 12,000 are performed. Although considered routine surgery with a low incidence of complications, there are certain situations when patient factors might make the surgery more difficult, such as previous surgery in the upper abdomen [1, 2].

Risk for gallstone formation is multifactorial; obesity is an independent risk factor for formation of gallstones, as is rapid weight loss, female gender, and pregnancy [3–6].

Obesity is increasing in the general population, including women of fertile age and during pregnancy [7–9]. In Sweden, the prevalence of obesity (defined as body mass index (BMI) > 30 kg/m^2^) in pregnant women has increased from 6.0% in 1992 to 14.1% in 2016 [10].

In the recent decades, bariatric surgery has become increasingly used as a mean to tackle the obesity pandemic and its comorbidities. An estimated number of 468,609 bariatric procedures were performed worldwide in 2013. In Sweden, the estimated prevalence of patients having had bariatric surgery was 63,000 in 2016 [11, 12]. A majority of these patients are females of reproductive age [13]. The rapid weight loss that occurs after bariatric surgery is thought to increase the risk of gallstone formation by accentuating other risk factors, leading to gallstone formation in 38% and sludge formation in 12% of patients [14].

Women are at higher risk of developing gallstones due to hormonal effects on bile composition. These effects are substantially increased in pregnancy, with sludge and gallstone formation as high as 31% and 2%, respectively [5].

With the development of minimally invasive techniques for gallbladder surgery, i.e., laparoscopic cholecystectomy, the risk of performing this procedure has now decreased [15]. In accordance with these findings, an increase in number of cholecystectomies in pregnancy has been observed in recent years [16].

Previous Roux-en-Y gastric bypass (RYGB) has been shown to be a risk factor for a markedly increased rate of complications after cholecystectomy, being doubled at 30-day follow-up, and a quadrupled risk of reoperation [17].

The aim of this study was to evaluate outcome for patients going through cholecystectomy in a rare, but growing, circumstance—ongoing pregnancy and previous bariatric surgery—compared with groups with only one or none of our two complicating parameters, i.e., pregnancy or previous bariatric surgery.

## Materials and Methods

Data for this study were extracted from the Swedish Registry of Gallstone Surgery and Endoscopic Retrograde Cholangiopancreatography (GallRiks) and the Scandinavian Obesity Surgery Registry (SOReg). Both these registries have a high nationwide coverage with 90% for GallRiks and 97% for SOReg and are validated continuously [18–20]. All adult female patients of fertile age (18–45 years) included in GallRiks from January 1, 2009, until March 12, 2016, were identified and combined with SOReg using the Swedish personal identity number. Patients with no information regarding pregnancy, duplicate entries, wrongly coded, and/or if the patient only underwent endoscopic retrograde cholangiopancreatography (ERCP) were excluded.

**Fig. 1:**
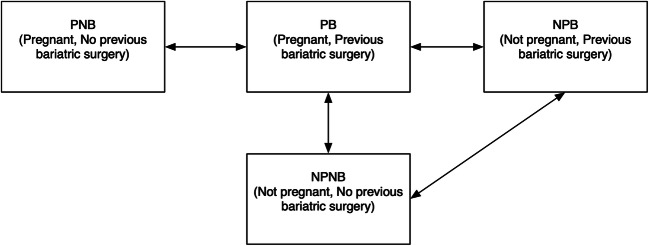
Comparisons made between groups

We defined four patient groups: pregnant with previous bariatric surgery (PB), pregnant with no previous bariatric surgery (PNB), non-pregnant with previous bariatric surgery (NPB), and non-pregnant with no previous bariatric surgery (NPNB). Further, we hypothesized that a majority of the pregnant patients had emergency surgery and that the majority of non-pregnant patients had planned surgery. To evaluate these groups, we divided the separate groups into subgroups with emergency procedures, henceforth called PB(E), PNB(E), NPB(E), and NPNB(E) (Fig. 1).

Baseline data were collected, including patient age and BMI and for the pregnant group their pregnancy week. Surgical data included indications for surgery (pancreatitis, cholecystitis, jaundice, or other), operative time, conversions from laparoscopic to open surgery, and postoperative length of stay (LOS). In addition to surgical time and LOS, intraoperative complications and postoperative complications at 30-day follow-up were used as outcome parameters. Perforated bowel, bile duct injury, bleeding requiring intervention, and other complications were registered as intraoperative complications. At 30-day follow-up, postoperative bleeding, deep infection, pancreatitis, postoperative bowel leak, cholangitis, thrombosis/emboli, biliary leakage, and biliary obstruction/jaundice were registered.

**Fig. 2:**
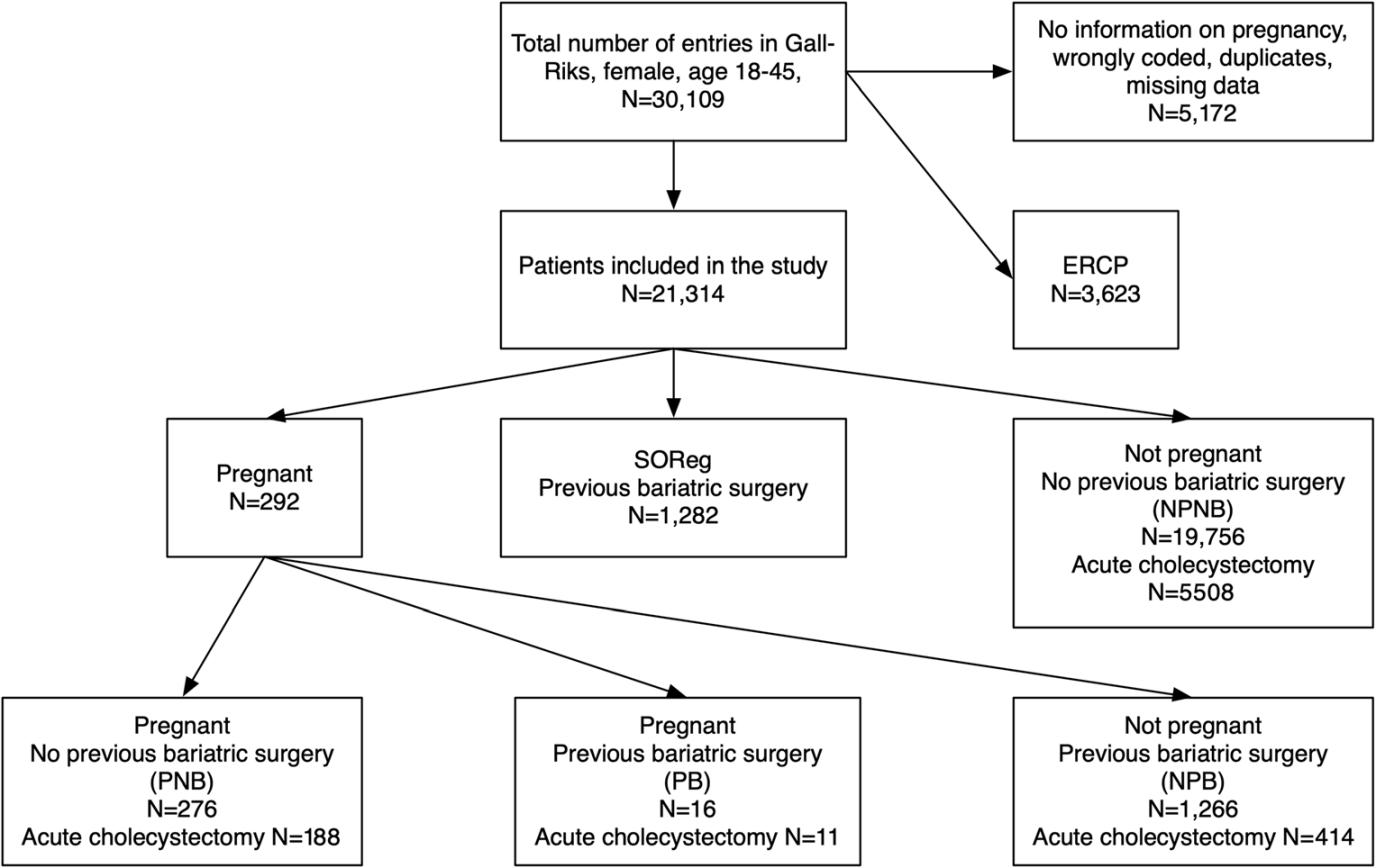
Flow diagram showing the number of patients extracted from the databases, and the division into different groups

The PB group were compared with the other three groups, and also PB(E) were compared with emergency surgical cases within the other groups. In addition, we compared the non-pregnant groups, NPB and NPNB, as well as NPB(E) and NPNB(E), for reference (Fig. 2).

### Statistical Analysis

The patients were described using absolute numbers, distribution in percentages, means, standard deviations (SD), medians, and interquartile ranges (IQR). Baseline characteristics between the patient groups were compared using Student’s *t* test, or Fishers exact test when expected frequencies were less than 5, and the unpaired Mann-Whitney *U* test for continuous variables, and the chi-square-test for categorical variables. Statistical analysis was made using the Stata MP, Version 4.2, statistical package for Mac OS X (Stata corporation LP, College Station, TX).

### Ethical Approval

The Regional Ethical committee in Lund approved the study (EPN Dnr 2014/177).

## Results

In total, 21,314 female patients aged 18–45 years that underwent cholecystectomy registered in GallRiks during our study period were included. Of these, 292 (0.99%) were pregnant at the time of cholecystectomy. When the patients in GallRiks were combined with SOReg data, 1282 patients with previous bariatric surgery were identified. The main study group: pregnant patients subjected to cholecystectomy that had a history of bariatric surgery (PB) numbered 16. A flow chart present extracted information from the databases, exclusion criteria, included patients, and the division into different groups (Fig. 2).

The PB patients had elective cholecystectomy in 5 cases and acute cholecystectomy in 11 cases. The indication for surgery was pancreatitis in one, jaundice in one, cholecystitis in three, and biliary colic in 11 patients. Laparoscopic technique was used in 14 and open surgery in 2. No patient in the PB group had intraoperative complications. One patient had a suspected port-site hernia, but no other complications were registered at 30-day follow-up. Choledocholithiasis was found in 3 of the PB patients: in one patient, intraoperative ERCP was performed; in one patient, open choledochotomy was performed; and in one patient, the stone could be flushed/manipulated to the duodenum using transcystic technique.

In the PB group, Roux-en-Y gastric bypass (RYGB) was the primary bariatric procedure in 15 patients, and one patient had biliopancreatic diversion with duodenal switch (DS) performed. Time interval between bariatric surgery and cholecystectomy was 104 days to 6.8 years (median 492 days). In the non-pregnant group, RYGB was performed in 1206 (96%) patients, DS in 13 (1%), and gastric sleeve in 38 (3%).

Both pregnant groups (PNB and PB) were compared. Significant longer LOS (2 [2, 3] days versus 1 [1, 2] days, *p* = 0.043) and operative time (108 (82–140 min) versus 83 (60–112) minutes, *p* = 0.031) were found in the PB group. When comparing the subgroups of PNB and PB having had emergency cholecystectomy, these differences were not seen. No difference in pregnancy week was seen between the groups and a majority of all pregnant patients had surgery in the second trimester (Tables 1 and 2).

**Table 1:** A comparison between pregnant patients without and with previous bariatric surgery, both elective and acute cholecystectomy cases are included. *n* (%) or median (IQR)

	*N* = 292	Pregnant No previous bariatric surgery *n* = 276	Pregnant Previous bariatric surgery *n* = 16	*P* value
Age	292	30 (25–34)	27 (26–36)	0.948
Pregnancy week	292	16 (10–20)	14 (12–20)	0.932
BMI	154	28 (24–33)	30 (25–32)	0.587
Acute cholecystectomy	292	188 (68%)	11 (69%)	1.000
Pancreatitis	292	30 (11%)	1 (6%)	1.000
Cholecystitis	292	59 (21%)	3 (19%)	1.000
Jaundice	292	35 (13%)	1 (6%)	0.703
Outcome				
Postoperative LOS (days)	277	1 (1–2)	2 (2–3)	0.043
Operative time (minutes)	292	83 (60–112)	108 (82–140)	0.031
Intraoperative complications	292	1 (0.4%)	0 (0%)	1.000
Conversion laparoscopic to open	292	3 (1%)	1 (6%)	0.201
Complications 30 days	276	25 (10%)	1 (7%)	1.000

*BMI* body mass index, *LOS* length of stay

**Table 2:** A comparison between pregnant patients with or without previous bariatric surgery, only acute cholecystectomy cases are included. *n* (%) or median (IQR)

	*N* = 199	Pregnant No previous bariatric surgery *n* = 188	Pregnant Previous bariatric surgery *n* = 11	*P* value
Age	199	30 (26–34)	32 (27–41)	0.242
Pregnancy week	199	16 (9–20)	14 (9–19)	0.588
BMI	99	28 (24–33)	32 (30–32)	0.288
Pancreatitis	199	28 (15%)	1 (9%)	1.000
Cholecystitis	199	56 (30%)	3 (27%)	1.000
Jaundice	199	34 (18%)	1 (9%)	0.693
Outcome				
Postoperative LOS (days)	187	2 (1–3)	2 (2–3)	0.421
Operative time (minutes)	199	88 (66–117)	121 (75–165)	0.125
Intraoperative complications	199	0 (0%)	0 (0%)	1.000
Conversion laparoscopic to open	199	2 (1%)	0 (0%)	1.000
Complications 30 days	186	21 (12%)	1 (11%)	1.000

*BMI* body mass index, *LOS* length of stay

For reference, we compared the non-pregnant women and found more acute cholecystectomy procedures in the group with previous bariatric surgery, longer LOS, longer operative time, more conversions from laparoscopic to open surgery, and an almost doubled incidence of complications at 30-day follow-up (Table 3). When comparing only emergency surgery for these both groups, we found the patients in the group without a previous bariatric procedure to be younger (33 (27–39) years versus 36 (29–40) years, *p* < 0.001). Further, the patients with previous bariatric surgery had longer LOS (2 [1–3] days versus 1 [1, 2] days, *p* < 0.001) and a higher incidence of 30-day postoperative complications (*n* = 59 (15%) versus *n* = 473 (9%), *p* < 0.001) but no difference could be seen in operative time (97 (70–132) minutes versus 100 (73–141) minutes, *p* = 0.068).

**Table 3:** A comparison between non-pregnant female patients of fertile age, with or without previous bariatric surgery, both elective and acute surgery cases are included. *n* (%) or median (IQR)

	*N* = 21,022	Not pregnant No previous bariatric surgery *N* = 19,756	Not pregnant Previous bariatric surgery *N* = 1266	*P* value
Age	21,022	34 (28–40)	35 (29–40)	0.286
BMI	13,910	28 (24–32)	28 (25–32)	0.145
Acute cholecystectomy	21,022	5508 (28%)	414 (33%)	< 0.001
Pancreatitis	21,022	593 (3%)	42 (3%)	0.498
Cholecystitis	21,022	2168 (11%)	141 (11%)	0.853
Jaundice	21,022	1348 (7%)	97 (8%)	0.252
Outcome				
Postoperative LOS (days)	20,255	1 (0–1)	1 (1–2)	< 0.001
Operative time (minutes)	21,021	83 (61–112)	90 (65–120)	< 0.001
Intraoperative complications	21,016	248 (1%)	19 (1%)	0.436
Conversion laparoscopic to open	21,022	437 (2%)	45 (3%)	0.004
Complications 30 days	20,249	1156 (6%)	132 (11%)	< 0.001

*BMI* body mass index, *LOS* length of stay

The incidence of acute cholecystectomy in our study group, PB, was significantly higher than the control group NPNB (11 (69%) vs 5508 (28%), *p* = 0.001). No differences in baseline characteristics or complications were found, but operative time and LOS were longer (Table 4). However, these differences were not seen when comparing only cases with an acute indication for surgery.

**Table 4:** A comparison between non-pregnant patients without previous bariatric surgery and pregnant patients with previous bariatric surgery, both elective and acute cholecystectomy cases are included. *n* (%) or median (IQR)

	*N* = 19,772	Not pregnant No previous bariatric surgery *n* = 19,756	Pregnant Previous bariatric surgery *n* = 16	*P* value
Age	19,772	34 (28–40)	27 (26–36)	0.058
BMI	13,002	28 (24–32)	30 (25–32)	0.528
Acute cholecystectomy	19,772	5508 (28%)	11 (69%)	0.001
Pancreatitis	19,772	593 (3%)	1 (6%)	0.386
Cholecystitis	19,772	2168 (11%)	3 (19%)	0.409
Jaundice	19,772	1348 (7%)	1 (6%)	1.000
Outcome				
Postoperative LOS (days)	19,055	1 (0–1)	2 (2–3)	< .0001
Operative time (minutes)	19,771	83 (61–112)	108 (82–140)	0.026
Intraoperative complications	19,766	248 (1%)	0 (0%)	1.000
Conversion laparoscopic to open	19,772	473 (2%)	1 (6%)	0.301
Complications 30 days	19,048	1156 (6%)	1 (7%)	0.584

*BMI* body mass index, *LOS* length of stay

When comparing the groups with previous bariatric surgery (NPB and PB), we found significant differences in age, acute cholecystectomy, and LOS (Table 5). However, when planned surgery was excluded, no differences could be seen.

**Table 5:** A comparison between pregnant and non-pregnant patients with previous bariatric surgery. Both elective and acute cholecystectomy cases are included. *n* (%) or median (IQR)

	*N* = 1282	Not pregnant Previous bariatric surgery *n* = 1266	Pregnant Previous bariatric surgery *n* = 16	*P* value
Age	1282	35 (29–40)	27 (26–36)	0.040
BMI	928	28 (25–32)	30 (25–32)	0.581
Acute cholecystectomy	1282	414 (33%)	11 (69%)	0.005
Pancreatitis	1282	42 (3%)	1 (6%)	0.423
Cholecystitis	1282	141 (11%)	3 (19%)	0.411
Jaundice	1282	97 (8%)	1 (6%)	1.000
Outcome				
Postoperative LOS (days)	1230	1 (1–2)	2 (2–3)	< 0.001
Operative time (minutes)	1282	90 (65–120)	108 (82–140)	0.091
Intraoperative complications	1282	19 (1%)	0 (0%)	1.000
Conversion laparoscopic to open	1282	45 (3%)	1 (6%)	0.444
Complications 30 days	1229	132 (11%)	1 (7%)	1.000

*BMI* body mass index, *LOS* length of stay

Intraoperative cholangiography is routinely performed during cholecystectomy in Sweden. In the pregnant groups, the frequency of successful cholangiography was significantly less than in the non-pregnant groups. No significant difference was seen in frequency in the PB and PNB groups (8 (50%) versus 99 (55%) *p* = 0.244).

## Discussion

Cholecystectomy in pregnant patients with previous bariatric surgery is still rare, even though the numbers are expected to steadily increase. By combining two validated national quality registries, we have been able to perform the first study of this specific patient group. The main finding is that cholecystectomy seems to be safe in the pregnant patient even if previous bariatric surgery has been performed. As expected, acute cholecystectomy is far more common than elective surgery in this group.

Strategies for handling asymptomatic and symptomatic gallbladder stones in patents eligible for bariatric surgery have been, and still are, debated. Higher complication rates in both concomitant and post-bariatric surgery cholecystectomy have been seen [17, 21–23].

Using the same registries as in our study, Wanjura et al. [17] noticed a doubled risk of postoperative complications after cholecystectomy and an almost quadrupled risk of reoperation in patients with previous Roux-en-Y gastric bypass. The higher risk of postoperative complications is confirmed in the present study for non-pregnant women in fertile age with a history of bariatric surgery. Other types of bariatric surgery, such as gastric sleeve procedures, were not included in Wanjuras study. There might be significant differences in complication rates depending on type of bariatric surgery. In our study, there were no significant differences in type of bariatric procedures performed between the groups and a vast majority were RYGB.

Laparoscopic cholecystectomy during pregnancy is considered safe and guidelines suggest that this is the treatment of choice in all trimesters [15]. There is, however, still concerns for both mother and fetus and the decision to perform surgery during pregnancy is not easy. If there is another risk factor present that would increase complication rates significantly, this decision would possibly be even more difficult. Low incidence of perioperative complications was seen in all our groups. Even if we confirmed the higher incidence of complications at 30-day follow-up in non-pregnant women in fertile age with a history of bariatric surgery, we did not find a higher risk for pregnant patients with a history of bariatric surgery. The reason for the better outcome in the pregnant population with previous bariatric surgery compared to the non-pregnant patients with previous bariatric surgery is unknown. It is reasonable to postulate that surgery during pregnancy implies a generally intensified effort to avoid complications, including surgery being performed by more experienced surgeons.

The vast majority of the pregnant patients in this study had their surgery performed in the second trimester. The reasons for this are unclear but may be due to that previous research, before the new guidelines, suggested the most favorable outcome in this time period [15, 24].

The indications for surgery in the pregnant population differ from the general population with a majority of procedures done as emergency surgery. This probably affects outcome parameters. When comparing some specific outcome parameters, such as LOS and operative time, the pregnancy itself can also influence the result. There might be reasons for a longer postoperative observation due to, e.g., extended fetal controls. Regarding operative time, technical issues such as port placement might affect operative time. Hence, longer LOS and operative time might be adequate consequences of a more careful approach to the pregnant patient as well as for non-pregnant patients with previous bariatric surgery.

New and rare patient groups and circumstances are hard to study. The strength of this study is the possibility to use validated registries to find and analyze outcome for a specific patient group, even if the low number still is a major limitation. Further, the study has limitations inherent to analysis of register data. Specific examples of limitations in these registries are lack of specification of the surgeon skill set, reasons for prolonged OT and LOS as well as no stratified registration of complications (e.g., Clavien-Dindo) [25].

In conclusion, even though cholecystectomy in fertile non-pregnant women with previous bariatric surgery imply an increased risk of 30-day complications, this increase in risk is not seen for the pregnant group with previous bariatric surgery. Our findings, with the proviso that the group is small, and conclusions drawn from such limited numbers must be viewed with caution, indicate that it is safe to perform cholecystectomy on pregnant patients who have had previous bariatric surgery. With an adequate indication for cholecystectomy, the same conclusion has been made for pregnant patients without prior bariatric surgery, where it according to guidelines is considered safe to perform surgery throughout the whole pregnancy period. More research is warranted considering the increasing number of pregnant patients with gallstone related problems with a history of bariatric surgery.
